# Improving Work Participation Outcomes Among Unemployed People with Mental Health Issues/Mental Illness: Feasibility of a Stigma Awareness Intervention

**DOI:** 10.1007/s10926-023-10141-3

**Published:** 2023-10-25

**Authors:** K. M. E. Janssens, M. C. W. Joosen, C. Henderson, J. van Weeghel, E. P. M. Brouwers

**Affiliations:** 1 Tranzo, Scientific Center for Care and Wellbeing, Tilburg School of Social and Behavioral Sciences, Tilburg University, P.O. box 90513, 5000 LE Tilburg, The Netherlands; 2 Department of Health Services and Population Research, King’s College London, London, UK; 3 Phrenos Center of Expertise, Utrecht, The Netherlands

**Keywords:** Process evaluation, Mental health issues/mental illness, Disclosure, Employment specialists, Vocational rehabilitation, Unemployed people

## Abstract

**Purpose:**

As stigma is a barrier to work participation of unemployed people with mental health issues/mental illness (MHI), a stigma awareness intervention can be helpful to make informed decisions about disclosing MHI. The aim of this process evaluation was to investigate the feasibility of a stigma awareness intervention, to explore experiences of clients and their employment specialists; and to give recommendations for further implementation.

**Methods:**

The intervention consisted of a stigma awareness training for employment specialists and a decision aid tool for their clients with (a history of) MHI. For the process evaluation, six process components of the Linnan & Stecklar framework were examined: recruitment, reach, dose delivered, dose received, fidelity and context. Using a mixed-methods design, quantitative and qualitative data were collected and analyzed.

**Results:**

The six components showed the intervention was largely implemented as planned. Questionnaire data showed that 94% of the clients found the tool useful and 87% would recommend it to others. In addition, more than half (54%) indicated the tool had been helpful in their disclosure decision. Qualitative data showed that participants were mainly positive about the intervention. Nevertheless, only a minority of clients and employment specialists had actually discussed the tool together. According to both, the intervention had increased their awareness of workplace stigma and the disclosure dilemma.

**Conclusion:**

The implementation of a stigma awareness intervention was feasible and did increase stigma awareness. Experiences with the intervention were mainly positive. When implementing the tool, it is recommended to embed it in the vocational rehabilitation system, so that discussing the disclosure dilemma becomes a routine.

**Trail Register:**

The study was retrospectively registered at the Dutch Trial Register (TRN: NL7798, date: 04-06-2019).

**Supplementary Information:**

The online version contains supplementary material available at 10.1007/s10926-023-10141-3.

## Introduction

### Background

People with mental health issues/mental illness (MHI) are 3 to 7 times more often unemployed than people without MHI [1, 2]. This is problematic, because being unemployed is associated with personal, social and economic consequences such as poorer (mental) health and financial strains [3–5] and under favourable conditions, employment contributes to health, wellbeing and recovery [1, 6]. There is growing evidence showing that stigma and discrimination are important barriers for the employment opportunities of people with MHI [7–10]. Stigma is the process of (negatively) labelling and excluding groups of people from society, which subsequently could lead to discriminatory behavior [11]. Link and Phelan’s theory [11] conceptualize stigma in four different components, i.e. (1) distinguishing and labeling human differences, (2) Cultural beliefs link these labels to undesirable characteristics and negative stereotypes; (3) Labels are placed in distinct categories to accomplish separation of ‘us’ from ‘them’; and (4) Because of labels, status loss and discrimination is experienced. Negative stereotypes and discrimination on the part of employers, as well as internalized stigma, i.e. turning the stigmatizing stereotypes to themselves, among people with MHI, could hamper finding and retaining paid employment [7, 12–16]. Furthermore, a lack of work experience could result in less return to work self-efficacy [17] and subsequently in the ‘why try’-effect (i.e. no longer participating in activities because of fear of discrimination) [8, 18].

Decisions about disclosure of a MHI to employers are complex and several studies have suggest that deliberate decisions are of importance for the (re-)employment success of people with MHI [7, 19–21], but are also complicated and personal [22]. Disclosure of MHI can lead to positive outcomes such as support or adjustments in the work environment [19], but could also have negative consequences such as stigma and discrimination (e.g. not getting hired) [19, 20]. A recent study found that the great majority of employees in the Netherlands had a strong preference to disclose MHI, as around 75% of Dutch employees indicated they had disclosed, or would want to disclose, their MHI to their manager [23, 24]. Studies have found that reasons for a disclosure preference are a positive relationship with the manager and high responsibility feelings towards the work environment [23–27]. However, 64% of Dutch managers were found to be reluctant to hire job applicants with MHI [13].

### Rationale

Decision aids for making informed decisions about whether or not disclosing MHI in the work context seem promising [21, 28, 29]. For example, the COnceal or ReveAL (CORAL) decision aid has shown to be effective in reducing decision-making stress, and in improving work participation of unemployed people with MHI in the UK [21]. Recently, the effectiveness of CORAL in combination with a stigma awareness training for employment specialists was tested in a randomized controlled trial (RCT) in Dutch municipal practice [30]. The intervention was found to be effective for unemployed people with MHI in finding and retaining paid employment. However, in addition to investigating the effectiveness of an intervention, it is also relevant to evaluate what elements contributed to the effectiveness, and to evaluate how the intervention was implemented in practice. Therefore, in this study, a process evaluation was conducted, in order to have a better understanding of the results of the RCT and the implementation of this intervention in the future.

### Aim

The aim of this process evaluation was to (1) investigate the feasibility of the stigma awareness intervention, (2) explore experiences of participants (i.e. clients and their employment specialists); and (3) give recommendations for further implementation of the stigma awareness intervention.

## Method

In this mixed methods study, data for the process evaluation was gathered alongside a cluster RCT, conducted between March 2018 and July 2020. For the process evaluation, 6 process components of the framework of Linnan and Steckler [31] were used: i.e. recruitment, reach, dose delivered, dose received, fidelity and context. Quantitative and qualitative methods were used to collect data on the process components among participants of the intervention group: questionnaires for clients and employment specialists, administrative data and telephone interviews with clients and employment specialists.

### Study Context

In the Netherlands, people who are above 18 years and have insufficient income or capital and who do not make use of other provisions or benefits (such as disability benefits), are entitled to social benefits. At the time of the study, 430.000–455.000 people received social benefits in the Netherlands [32]. Of the people receiving social benefits, 31% receive mental health care [33]. Taken into account the treatment gap among people with MHI [34], this is likely to be an underestimation of the actual percentage of people with MHI who receive social benefits. Receiving social benefits involves specific rights and obligations through the *Work and Social Assistance Act* (2004). One of these obligations is cooperating in the support municipalities offer, aimed at entering the job market or returning to existing employment. Municipalities are authorized to organize this support by themselves. As a result, some have their own vocational rehabilitation services and employment specialists who provide vocational rehabilitation to (often long-term unemployed) clients receiving social benefits from the municipality, whereas other municipalities hire the services of external organizations. The support could be organized as one-on-one appointments or as job application group training sessions to the clients.

### Study Population

In the cluster RCT [30], eight organizations (i.e. municipalities and organizations who work on behalf of these municipalities) participated. Organizations were reached via the networks of the researchers. Subsequently, the researchers presented the study design during meetings at the local organizations. Randomization took place on organization level (i.e. cluster randomization). Four organizations were randomized to the intervention group and four organizations to the control group. In this study, only the organizations of the intervention group are included.

The intervention focused on 2 groups of participants: (1) unemployed people with MHI receiving social benefits, hereafter *clients* and (2) employment specialists working at the local municipalities who provided clients with guidance to find paid employment, hereafter *employment specialists*.

#### Clients

*N* = 76 clients participated in the intervention group of the study. Inclusion criteria were (1) being unemployed, i.e. an income below minimum income and receiving social benefits; (2) self-reporting to have either had a current MHI (including addiction), or to have had MHI in the past and having sought any treatment (currently or in the past) for that by a health professional (e.g. general practitioner, psychiatrist, psychologist). Type or severity of the MHI was irrelevant for inclusion in the study; and (3) adequate command of the Dutch language, as the intervention and questionnaires were in Dutch. Clients filled out questionnaires at four measurements (baseline, 3, 6 and 12 months) with questions about personal characteristics and questions regarding the feasibility of CORAL.NL (i.e. Dutch version of CORAL decision aid, see Online Appendix 1 and 4 for Dutch version and Online Appendix 2 for English version). Clients received a financial remuneration of 10 euros after filling out each questionnaire (in total clients could receive 40 euro for completing all questionnaires) to motivate them to complete the longitudinal study and to thank them for their time.

After completing the intervention study, clients of the intervention group were invited by the researchers for a telephone interview. The invited clients were a representation of the total sample in age, gender, educational level and did/did not found paid employment during the study period. *N* = 7 clients were not reached, other reasons for not participating in the interviews were: not interested (*N* = 4), too busy (*N* = 3), personal reasons (*N* = 1), and did not remember participating in the intervention study (*N* = 1). After interviews with *N* = 16 clients data saturation was reached. All signed an informed consent prior to the interview. Clients received a financial remuneration of 10 euros to thank them for their time.

#### Employment Specialists

Participating employment specialists were working at one of the four organizations of the intervention group, i.e. municipalities and organizations who work on behalf of municipalities. The employment specialists provided vocational rehabilitation to the clients who participated in this process evaluation and received the stigma awareness training for employment specialists, which was part of the intervention. In total, self-report data from *N* = 35 employment specialists was used. Employment specialists filled out questionnaires at three measurements (prior to stigma awareness training and directly and 12 months after stigma awareness training) with questions about personal characteristics and questions regarding the feasibility of the stigma awareness training and CORAL.NL.

In addition, after completing the intervention study, those employment specialists of the intervention group that who were still working at their organization (*N* = 13) were invited by the researchers for a telephone interview. Of them, *N* = 12 responded positively and signed an informed consent to participate in the interview. One employment specialist was not willing to participate in the interview.

### Intervention

The stigma awareness intervention had two elements: (1) a printed booklet of the decision aid CORAL.NL for people with MHI and two infographics, i.e. simplified versions of the decision aid for those with literacy or concentration problems and (2) a 3 × 2 h training targeted at employment specialists to increase their awareness about workplace stigma. In the RCT, the control group received vocational rehabilitation without the stigma awareness intervention, i.e. practice as usual.

#### CORAL.NL Tool

The CORAL.NL decision aid is based on the English Conceal Or ReveAL (CORAL) decision aid [21]. The decision aid was translated and developed further into the CORAL.NL for the Dutch practice by conducting a focus group study [19]. Adjustments were related to Dutch legislation (i.e. Dutch employers are not allowed to ask questions about an employee’s illness), and by including more information about the disclosure process (i.e. who to disclose to, timing, preparation, message content and communication style) as these topics were found to be important in a previous focus group study [19]. The CORAL.NL decision aid entails a printed booklet consisting of four parts with several paragraphs: (1) choices about disclosure, including pros and cons of (non-)disclosure (e.g. ‘*You can ask your employer for time off to go to things like doctor’s appointment*’ and ‘*You may be less likely to get the job*’) and personal needs and values; (2) identifying the personal situation, including preferences about when and to whom to disclose; (3) tips (e.g. emphasize on telling what you need to do your job well rather than mentioning the mental health diagnosis, practice how to (not) disclose with a trusted one); and (4) a recap of previous sections to make a plan about whether and what to disclose or not, and if so, to whom and when. When pilot tested by employment specialists who worked with people with MHI, the CORAL.NL (a 14-pages booklet) was seen as too elaborate for people with lower concentration or reading skills. Therefore, two one-page infographics were created as a brief summary of the CORAL tool: one version about disclosure during the job application process and the other about disclosure during employment (see Online Appendix 1). The infographics consisted of three parts, including (1) reasons not to disclose MHI, (2) reasons to disclose MHI and (3) some tips about what to disclose (e.g. emphasize on telling your needs to do your job well). For the infographics, only a few items from the CORAL tool could be chosen. The selected items were confirmed to be important in a prior study on workplace mental health disclosure [19].

#### Stigma Awareness Training for Employment Specialists

Employment specialists participated in a stigma-awareness training about disclosure of MHI in the work context, specifically designed for the purpose of this study. While developing the training, input from a focus group study was used [19] combined with recent literature about working elements in destigmatizing interventions [35–37]. Important working elements are education about (people with) MHI and social contact between people with and without MHI in a context of equality [35]. Therefore, these elements were included in all training sessions. Specifically, the first training session entailed a live interview with a mental health advocate with lived experience, followed by an interactive discussion. In the second training session, a short film with personal stories of five workers with MHI who had experienced workplace stigma and discrimination was shown and discussed. In the final training session, employment specialists practiced conversations about the disclosure dilemma with a mental health advocate with lived experience. Aims of the training were enhancing awareness for (1) mental health workplace stigma and discrimination; (2) the disclosure dilemma; and (3) practice use of CORAL.NL and enhance skills for implementation. An overview of the learning goals and format of the training sessions is shown in Online Appendix 2.

The training consisted of three meetings of two hours, guided by 2–3 researchers (KJ and EB and/or MJ) and were provided in groups of 4–12 employment specialists at their own organizations. During the first meeting, employment specialists were trained to start to work with CORAL.NL in practice. In the training sessions, employment specialists were stimulated and reminded to use the CORAL.NL tool in practice, after clients had completed the baseline questionnaire.

### Data Collection

#### Feasibility of the Intervention

To examine the feasibility of the intervention, the framework of process components by Linnan and Steckler [31] was used. The process components were described on the level of (a) clients and (b) employment specialists.

- Recruitment: The procedures used to approach participants for the intervention. The recruitment of both clients and employment specialists was described.
- Reach: The proportion of the intended target group that participated in the intervention. For clients, reach was defined as the proportion of those who actually participated in the study divided by the number of clients that were reached by the various recruitment strategies. For employment specialists, reach was the proportion that participated in the intervention group divided by the number of employment specialists that were invited to participate.
- Dose delivered: The number of intended interventions that is actually delivered. In the present study dose delivered was defined for clients as the number who received the CORAL.NL tool (i.e. the booklet and infographics) by the intervention providers. For employment specialists, dose delivered is the proportion that attended the training meetings according to the protocol.
- Dose received: The extent to which participants engaged in the intervention. For clients, dose received was defined as the proportion that (1) has read the intervention, and (2) has discussed the content of the CORAL.NL tool with their employment specialist. For employment specialists, dose received is the proportion that participated in the training meetings, and the proportion that indicated in (open) questions to actively work with the CORAL.NL tool which was introduced in the training (i.e. ‘Did you use the CORAL.NL booklet/infographics in supporting clients with MHI?’, ‘Why did you use the CORAL.NL tool?’ and ‘Do you still use the CORAL.NL tool in supporting clients with mental health issues/mental illness?’).
- Fidelity: The extent to which the intervention was implemented and delivered as planned. For clients, fidelity was defined as the extent to which the CORAL.NL tool was implemented as planned, i.e. as a tool for clients and employment specialists to think more deliberate about disclosing MHI and/or have a conversation about the disclosure dilemma. For employment specialists, fidelity is the extent to which the training meetings were delivered as planned, and the extent to which the CORAL.NL was implemented in their support to clients. This was evaluated by self-report data from clients about their disclosure decisions and attitudes towards the CORAL.NL tool. Attitudes towards the perceived utility of the CORAL.NL tool were measured using eight statements (e.g. ‘I believe the CORAL.NL infographics were useful’, ‘The CORAL.NL tool has played an important role during the application process’ and ‘I would recommend the CORAL.NL tool to others’) with four answer categories: totally disagree, disagree, agree, totally agree. In this study, totally disagree and disagree were merged into ‘disagree’, totally agree and agree were merged into ‘agree’. In addition, data from telephone interviews with both clients and employment specialists were used.
- Context: Aspects of the environment that may have influenced the implementation of the intervention. Both the context for clients and employment specialists will be described. The process component context was assessed by telephone interviews.

#### Telephone Interviews with Clients and Their Employment Specialists

Telephone interviews were held with clients and employment specialists to collect qualitative data for the process components *fidelity* and *context*. Prior to the interviews, two topic lists (one for clients and one for employment specialists) were developed based on the research questions of this study and the framework of process components [31]. The topic lists consisted of questions about experiences regarding feasibility, working elements and effects of the intervention on finding and retaining paid employment, and on what experienced barriers and facilitators were for a successful implementation. Telephone interviews lasted for about 15–30 min.

### Data Analysis

Data of the questionnaires were analyzed using descriptive statistics. These statistical analyses were performed using SPSS version 25.0 for Windows. Interviews with participants were digitally recorded and transcribed verbatim. Transcripts were anonymized before analyses were performed. Interviews were coded and categorized through thematic coding by researcher KJ, using the qualitative data analysis software program Atlas.ti, version 9. Researchers EB, MJ and JW each checked the coding of two interviews (one of clients and one of employment specialists). Code agreements and disagreements were discussed within the team. Disagreements were reconsidered until agreement was reached.

## Results

Mean age of clients was 37.4 years, and 58% was female. Most frequent self-reported psychiatric diagnoses were depression (26%), autism spectrum disorder (18%) and burnout (16%). For employment specialists, the mean age was 42.7 years and 84% was female. The mean years of work experience was 17.2 years, and the mean years of experience working with clients with MHI was 7.7 years (see Table 1).

**Table 1:** Characteristics of the research sample

	M (SD)/*N* (%)
Clients ( *N* = 76)	
Age	37.4 (11.9)
Gender: male	32 (42%)
Marital status: no relationship	62 (82%)
Education level	
Lower educated or no education	39 (51%)
Medium educated	24 (32%)
High educated	13 (17%)
Self-report diagnosis^a^	
Anxiety	6 (8%)
Attention deficit (hyperactivity) disorder	11 (15%)
Autism spectrum disorder (including Asperger and PDD-NOS)^b^	14 (18%)
Bipolar disorder	2 (3%)
Burnout, stress, overload	12 (16%)
Depression	20 (26%)
Personality disorder	11 (15%)
Psychotic disorder	3 (4%)
Posttraumatic stress disorder	12 (16%)
Other diagnosis	7 (9%)
Don’t know diagnosis	7 (9%)
No official diagnosis	11 (15%)
Have had employment before baseline: yes	72 (95%)
Employment specialists (N = 35)	
Age	42.7 (8.1)
Gender: male	8 (16%)
Years of work experience	17.2 (7.9)
Years of experience working with people with mental health issues/mental illness	7.7 (5.7)

^a^*N* = 38 participants (50.0%) had comorbidity (i.e. two or more diagnoses)

^b^Pervasive developmental disorder-not otherwise specified

### Recruitment

*Clients* were recruited through the four participating organizations. Employment specialists personally asked eligible clients if they were willing to receive more information about the study by telephone by the researchers. However, this recruitment strategy did not ensure enough eligible clients. Therefore, eligible clients were also recruited via personal invitation letters and leaflets from the organizations where the participating employment specialists were employed. Table 2 gives an overview of the number of clients recruited via the various recruitment strategies.

**Table 2:** Recruitment strategies and number of clients that were reached for participating in the RCT (intervention group)

Recruitment strategies	Recruited^a^	Willing to have an introduction by telephone^a, b^	Reached^a,b,c^	
	(*N*)	(*N*)	(*N*)	(%)
Recruited by employment specialists				
In one-by-one contact	88	77	59	59/88 = 67%
During job application training sessions	20	7	5	5/20 = 25%
Personal letter or email from the municipality/organization	320	21	12	12/320 = 4%
Leaflets in waiting rooms of municipality/organization	Unknown	0	0	0%
Total	Minimum of 428	105	76	76/428 = 18%

^a^Recruited* = *eligible clients that were recruited to participate in the RCT

^b^Willing to have an introduction by telephone = eligible clients who gave consent to be contacted by researchers for more information about participating in the RCT

^c^Reached = intended target group that actually participated in the intervention

*Employment specialists* were recruited within the four participating organizations. Two small organizations working in small teams (circa 8 employment specialists) invited all their employment specialists to participate in the study. In one large organization, the manager of a large team invited a selection of employment specialists who were not already involved in other projects or studies. In the other large organization, the manager selected the employment specialists of their team, as there were also other professionals (i.e. social workers, debt counselors) involved in their teams.

### Reach

After being asked by employment specialists to be willing to receive more information about the study from the researchers, *clients* were contacted by telephone by the researchers to give this information, check the inclusion criteria and to invite to participate. Here, information was provided, including that their decision whether or not to participate had no consequences for their contacts with their employment specialist, and that participation was entirely on a voluntary basis and anonymous. With some recruitment strategies (e.g. personal letters), clients who did not meet the inclusion criteria (e.g. not having (had) MHI) were also recruited, but they were excluded from participating in the study. Furthermore, clients may have been recruited in two or more ways (e.g. via the employment specialist and via a personal letter). The reach percentages for the recruitment strategies were: 59/88 = 67% for personal invitations by employment specialists, 5/20 = 25% for recruitment during job application training sessions, 12/320 = 4% for invitations via personal letter or email from the organizations, and 0/0 = 0% for leaflets in waiting rooms of the organizations. The reach percentage for all recruitment strategies together was 76/428 = 18% (see Table 2).

For *employment specialists*, the reach percentage was 100% for two small organizations (*N* = 17). For one large organization, ten employment specialists were invited by their team manager to visit an information session about the research. After the sessions, eight employment specialists were willing to participate, therefore the reach percentage was 8/10 = 80%. Within the other large organization, eight employment specialists were reached by their team managers and willing to participate (8/8 = 100%). The total reach percentage was 33/35 = 94%.

### Dose Delivered

All *clients* received the CORAL.NL booklet and infographics from the researcher after filling out the baseline questionnaire. This resulted in a dose delivered of 100%.

For *employment specialists*, all of them (*N* = 35, 100%) participated in the first training session. *N* = 7 employment specialists dropped out after the first training session because of several reasons: not willing to participate in the study anymore (*N* = 3), not working in the organization anymore (*N* = 3) and maternity leave (*N* = 1). After the second training session, *N* = 8 employment specialists dropped out (not working in the organization anymore: *N* = 7, not willing to participate in the study anymore: *N* = 1). In total, *N* = 20 employment specialists (57%) completed the full training.

### Dose Received

After filling out the baseline questionnaire, clients received the CORAL.NL tool by the researchers. Although employment specialists were instructed not to hand out the tool to clients before their participation at baseline, N = 3 clients (4%) had received the tool from their employment specialist prior to baseline (data not shown in table). As can be found in Table 3, 3 months after baseline, 59% of the *clients* indicated in the questionnaires that they were familiar with the tool. Respectively, after 6 and 12 months, 61% and 69% of the clients were familiar with the tool. The CORAL.NL infographics had been read by 71% of the clients, and the booklet by 65% of the clients after 12 months. Around 16–18% of the clients discussed the tool with their employment specialist during the study period (see Table 3).

**Table 3:** Clients’ use and experiences with the CORAL.NL tool

	*T*1 3 months (*N* = 67)	*T*2 6 months (*N* = 67)	*T*3 12 months (*N* = 65)
I am familiar with the CORAL.NL tool	41 (61.2%)	41 (61.2%)	45 (69.2%)
I have read the CORAL.NL infographics	37 (55.2%)	37 (55.2%)	46 (70.8%)
I have read the CORAL.NL booklet	35 (52.2%)	39 (58.2%)	42 (64.6%)
I have discussed the CORAL.NL tool with their employment specialist	13 (19.4%)	11 (16.4%)	11 (16.9%)
Statements about the CORAL.NL tool^a^			
I believe the CORAL.NL infographic is useful	34 (82.9%)	39 (90.7%)	45 (93.8%)
I believe the CORAL.NL booklet is useful	34 (82.9%)	39 (92.9%)	43 (91.5%)
I have benefited a lot from the CORAL.NL tool	20 (54.1%)	22 (51.2%)	21 (44.7%)
The CORAL.NL tool has played an important role during the application process	9 (24.3%)	10 (23.3%)	10 (21.7%)
The CORAL.NL tool has played an important role in finding paid employment	6 (16.2%)	10 (23.8%)	10 (21.3%)
The CORAL.NL tool has helped me in deciding whether or not disclosing my mental health issues/mental illness to an employer	23 (59.0%)	23 (54.8%)	26 (55.3%)
The CORAL.NL tool has changed my mind about disclosure of mental health issues/mental illness	26 (53.1%)	17 (40.5%)	21 (44.7%)
I would recommend the CORAL.NL tool to others	35 (85.4%)	36 (85.7%)	41 (87.2%)

Clients received the CORAL.NL tool after filling out the baseline questionnaire

^a^Only answered by clients who have read the CORAL.NL infographics and/or booklet in the former 3 months

After completing the stigma awareness training, 68% of *employment specialists* indicated they had used the CORAL.NL infographics and 26% had used the CORAL.NL booklet in their contact with clients with MHI. Employment specialists who indicated they used the tool did not use these during every client contact. Of the employment specialists who used the tool (*N* = 13), six indicated to use them ‘because of the importance of the topic’, three ‘because clients asked questions about disclosure’ and three for other reasons. 1 year after the training, 41% reported still using the infographics and 26% the booklet. Of the employment specialists who reported using the tool, only one specialist used the tool during every client contact. Of the employment specialists who still used the tool (*N* = 11), one reported using them ‘because of the importance of the topic’, three ‘because clients asked questions about disclosure’ and three for other reasons (see Table 4).

**Table 4:** Percentage of employment specialists that actively engaged with the intervention (*N* (%))

	Immediately after completing stigma awareness training (*N* = 19)	1 year after completing stigma awareness training (*N* = 27)
Did you use the CORAL.NL infographics in supporting clients with mental health issues/mental illness	13 (68.4%)	11 (40.7%)
Did you use the CORAL.NL booklet in supporting clients with mental health issues/mental illness	5 (26.3%)	7 (25.9%)
Why did you use the CORAL.NL tool?^a^		
Because of the importance of the topic	6 (46.1%)	1 (9.1%)
Because clients asked questions about disclosure	3 (23.1%)	3 (27.2%)
Other	3 (23.1%)	3 (27.2%)
Unknown (did not answer)	1 (7.7%)	5 (45.4%)
Do you still use the CORAL.NL tool in supporting clients with mental health issues/mental illness?		
Yes, always	0 (0.0%)	1 (3.7%)
Yes, sometimes	10 (52.6%)	6 (22.2%)
No	9 (47.4%)	20 (74.1%)

^a^Only answered by clients who have read the CORAL.NL infographics and/or booklet in the former 3 months

### Fidelity

*Clients* received the CORAL.NL tool, i.e. a decision aid to make more deliberate disclosure decisions in the work context, after filling out the baseline questionnaire. In case clients lost the tool or did not remember it anymore at follow-up questionnaires, the tool was provided again. In the questionnaires was found that after 12 months, 94% of the clients indicated that they believe the CORAL.NL infographic was useful, and 92% of the clients believed the CORAL.NL booklet had been useful. The CORAL.NL tool was recommended to others by 87% of the clients. For 54% of the clients the tool was helpful in deciding whether or not disclosing their MHI to an employer, and 45% indicated that the tool had changed their mind about disclosure of MHI. About one in five (22%) of the clients indicated that the tool had played an important role during their job application process and 21% indicated that the tool had been important during finding paid employment (see Table 3). In the interviews, most clients mentioned they believed that discussing the tool and the disclosure decision with their employment specialist would have been useful, although they had not discussed it.

*Employment specialists* were asked by managers to participate in the study. Participating in the study as employment specialist was voluntary and employment specialists filled out informed consent forms. Although the employment specialists were required by their managers to attend the training sessions, they were free to do what they wanted with the knowledge from the stigma awareness training. In addition, employment specialists were motivated but not obligated to recruit participants for the study. Employment specialists’ training sessions were provided at their organizations. If an employment specialist could not be present at a training session, a separate training session (alone or together with other employment specialists who could not be present) was organized. In the interviews, employment specialists mentioned that through the training sessions, the topic of disclosure had become more part of the conversation with clients with MHI. Employment specialists experienced more awareness about the disclosure dilemma and the everyday presence of stigmas because of the training sessions. Employment specialists mentioned to use the tool especially with clients who were actively searching for work and not to use it with clients who would deny their MHI because of having a non-Western cultural background and therefore having very different views on what MHI are, had concentration or literacy problems or were not ready to search for work yet. One of the most appreciated aspects by employment specialists was the presence of a mental health advocate with lived experience during the training sessions, which had impressed them. Furthermore, employment specialists mentioned that they had appreciated the presentations on scientific research of workplace stigma and the disclosure dilemma and the interactive debates about topic related statements, and had found these to be informative.

### Context

*Clients* did not always have frequent meetings with their employment specialist, e.g. because employment specialists could postpone appointments in case they estimated the MHI at that moment as too severe, which hindered discussing the CORAL.NL tool with their employment specialists. In the interviews clients were asked about their opinion of the feasibility of the CORAL.NL tool. Clients found the CORAL.NL tool clear and well structured, with good explanations. Some clients mentioned that they were not yet actively seeking for a job and therefore did not see the importance of thinking whether to disclose or not. Other clients sometimes distrusted their employment specialist, thinking they were only trying to get them to work because that would save the municipality money, rather than being interested in and supportive of clients’ well-being. Facilitators mentioned for the use of the CORAL.NL tool was having a good relationship with their employment specialist and having an employment specialist who was interested in the disclosure dilemma.

In the interviews, the majority of the *employment specialists* mentioned that working with the tool had not become a routine and that using the tool was not necessary to discuss the disclosure dilemma with clients. However, the disclosure dilemma had become a more prominent topic of discussion in their contact with clients. They indicated that it would have helped if they would have been reminded more often to use the tool by the researchers. *‘Yes, I still have it in my mind, but it does fade away. [Also because I see a lot of clients], so it sometime [the appointments] goes quite quickly, and I notice in myself that when a lot of new things come up, certain things also recede into the background’ (Professional)* In addition, employment specialists indicated that more frequent contact with the researchers and/or more training sessions could have been a facilitator to maintain focus on the disclosure topic. Employment specialists reported in the interviews that the content of the training quickly became of minor importance in their guidance of clients because of other tasks and work activities.

#### Previous Disclosure Experiences and Experiences Regarding Participating in the Intervention

At the baseline measurement, of the *clients* who had applied for work, 12% of the clients had disclosed their MHI in some job application letters, and 23% of the clients had disclosed their MHI sometimes or always during a first job application interview. After 12 months, none of the clients had disclosed their MHI in a job application letter and 19% of the clients had disclosed their MHI in a first job interview (see Table 5).

**Table 5:** Frequencies of clients’ disclosure decisions in the work context (*N* (%))

	*T*0 Baseline (*N* = 75)	*T*1 3 months (*N* = 67)	*T*2 6 months (*N* = 67)	*T*3 12 months (*N* = 65)
In the past 4 weeks, did you disclose your MHI in a job application letter?				
Never	29 (38.7%)	30 (44.8%)	33 (49.3%)	28 (43.1%)
Sometimes	4 (5.3%)	2 (3.0%)	1 (1.5%)	0 (0%)
Always	0 (0.0%)	1 (1.5%)	1 (1.5%)	0 (0%)
Not applicable (i.e. did not write a job application letter)	42 (56.0%)	34 (50.7%)	32 (47.8%)	37 (56.9%)
In the past 4 weeks, did you disclose your MHI in a first job application interview?				
Never	25 (33.3%)	27 (40.3%)	28 (41.8%)	22 (33.8%)
Sometimes	7 (9.3%)	5 (7.5%)	3 (4.5%)	5 (7.7%)
Always	1 (1.3%)	3 (4.5%)	3 (4.5%)	0 (0%)
Not applicable	42 (56.0%)	32 (47.8%)	33 (49.3%)	38 (58.5%)
In the past 4 weeks, did you disclose your MHI in a follow up job application interview?				
Never	19 (25.3%)	24 (35.8%)	25 (37.3%)	21 (32.3%)
Sometimes	6 (8.0%)	6 (9.0%)	4 (6.0%)	4 (6.2%)
Always	0 (0.0%)	1 (1.5%)	2 (3.0%)	1 (1.5%)
Not applicable	50 (66.7%)	36 (53.7%)	36 (53.7%)	39 (60.0%)
In the past 4 weeks, did you disclose your MHI after being hired?				
Never	15 (20.0%)	21 (31.3%)	21 (31.3%)	21 (32.3%)
Sometimes	9 (12.0%)	11 (16.4%)	7 (10.4%)	6 (9.2%)
Always	1 (1.3%)	1 (1.5%)	4 (6.0%)	0 (0.0%)
Not applicable	50 (66.7%)	34 (50.7%)	35 (52.2%)	38 (58.5%)

In the interviews, *clients* indicated that information about (non-)disclosure decisions was useful. They reported that increasing awareness of the disclosure dilemma was an important effect of the CORAL.NL tool. Clients said that as a result of the CORAL.NL tool they had become more aware of the pros and cons of both disclosure and non-disclosure. ‘*Well, especially the idea of having a choice has really helped me, you know. I never really thought about it before. There’s absolutely no obligation to share those things; it’s more like… it can be helpful to share them’ (Client).* In some cases, clients retained their original disclosure decision, however this decision was now more deliberate rather than intuitive only. Other clients reported that they had changed their mind after using the tool, especially from disclosure to non-disclosure but also from non-disclosure towards disclosure.

Most employment specialists were motivated to participate in the training sessions and reported that they had become more aware about stigma of MHI and the disclosure dilemma. Some of the interviewed *employment specialists* mentioned that they had hoped to learn more about how to deal with and support clients with MHI in their vocational rehabilitation and were somewhat disappointed that the stigma awareness intervention had not addressed this.

## Discussion

The aim of this process evaluation was to investigate the feasibility of a stigma awareness intervention, to report experiences of clients and their employment specialist, and to give recommendations for further implementation in practice. The stigma awareness intervention consisted of the Dutch CORAL.NL decision aid and a newly developed stigma awareness training for employment specialists. The overall results show that the intervention was feasible to implement and that the intervention proved to be successful in increasing stigma awareness and awareness about the disclosure dilemma in both clients and their employment specialists.

The results of the study showed that the majority of the clients were positive about the content of the CORAL.NL tool. Clients affirmed they had become more aware about the importance of deliberate disclosure decisions and most of the clients would recommend the tool to others. In addition, the tool was reported to be helpful for the majority of the clients in making a decision about whether to disclose MHI or not, and 40–53% of the clients had changed their mind about disclosure of MHI due to the tool. About one in five clients indicated that the tool had helpful in applying and/or finding work. This suggests that the timing of presenting the tool to clients may be important, where it is more helpful for those people who are actively searching and/or applying for work [21]. Another explanation may be that the tool makes people feel more empowered, which may reduce self-stigma and increase someone’s self-esteem [38, 39]. Subsequently, this could lead to more positive work participation outcomes.

Results of a separately conducted RCT examining the effects of current stigma awareness intervention have shown that participants of the intervention group had found (51%) and retained (49%) paid employment twice as often compared to the control group (respectively 26% and 23%) [30]. In addition, participants of the experimental group reported to be more satisfied with the support received from their employment specialists [30]. This illustrates that it is highly important to educate and motivate employment specialists in using such interventions the tools and address the topics of stigma and disclosure with their clients. In times of high unemployment rates, which has increased only more after the COVID-19 pandemic and especially for people with MHI [40–42], this intervention may substantially contribute to improved employment opportunities of people with MHI, have great financial implications on a societal and personal level.

Concerning the stigma awareness training, most employment specialists adhered to completing all training sessions. Employment specialists’ opinions about the training sessions were divided. Most (teams of) employment specialists were very enthusiastic and motivated to participate in the training sessions, whilst others did not see added value. Employment specialists mainly dropped out the training sessions because of job changes. However, four employment specialists dropped out because they lost interest to participate in the study. Perhaps, this may be the result of some employment specialists’ disappointment about the content not being more broadly about how to help people with MHI. Effective elements in stigma awareness interventions are face-to-face contact with someone with lived experience and the educative components [35, 43–45], and these were also present in the current stigma awareness intervention and much appreciated by the employment specialists. However, in further implementation, having trainers with an employment specialist background providing the training sessions might increase participation of employment specialists because they better able to respond to the needs of employment specialists from their personal experiences.

In this process evaluation, six process components of Linnan and Steckler’s framework [31] were explored. Of all strategies, recruitment via personal invitations from employment specialists had the highest reach percentage. Other strategies (such as invitations via personal letters or email) had a lower reach percentage but were less time intensive and included in total more eligible clients. Recruitment of clients via employment specialists can cause difficulties because of keeping them involved and motivated to recruit [46]. For this reason, in this study other recruitment strategies were needed. In addition, recruitment via employment specialists could create selection bias [47], e.g. employment specialists who prevent their clients from participating or because they were unaware of the clients’ MHI because the client did not disclose.

This process evaluation has shown that the intervention was largely implemented and conducted as planned. However, the adherence to the intervention by clients and employment specialists could have been better. Low adherence to interventions is a problem in many studies (e.g. [48–50]), as in the current study. Around two third of the clients had read the CORAL.NL tool and one fifth of the clients had discussed the CORAL.NL tool with their employment specialists. For employment specialists, after completing the training sessions, half of them used the tool during some of their client contact. After 1 year, a quarter of the employment specialists still used it (sometimes). Improving the adherence of the intervention by clients and employment specialists in future implementation may even improve the effectiveness of the intervention on employment outcomes. Therefore, it might be helpful to systematically embed the CORAL.NL into vocational rehabilitation services. This may ensure that the tool is accessible to everyone who wants to, as the tool was not always at the forefront of employment specialists’ minds. Currently, in the Netherlands, practitioners of the supported employment method Individual Placement and Support have already incorporated the tool into their guidance.

### Strengths and Limitations

A strength of this process evaluation is the use of the theoretical framework of Linnan & Steckler [31]. Using a theoretical framework ensures several relevant process components are assessed thoroughly. Second, a strength of the current study is the use of both quantitative and qualitative data, as well as the combination of data from clients together with data from their employment specialists. A limitation of this study is the lack of a fidelity instrument to measure the feasibility of the stigma awareness intervention in a structured fashion. Another limitation was the lack of information available from eligible clients who decided not to participate in the study or who were not invited by their employment specialists to participate in the study. Therefore, it was not possible to conduct a non-response analysis. The municipalities’ vocational rehabilitation services support all unemployed people in finding employment. Although people in a severe and acute phase of mental illness were not excluded from the study, they may not have been seen as eligible for the study (and/or for vocational rehabilitation) by the employment specialists. It is remarkable that in current study few clients with an anxiety disorder participated, despite the fact that the proportion of anxiety disorders among MHI is much higher, which could create bias in current study. A possible explanation may be that individuals with anxiety disorders are less inclined to participate in this type of research. Future research should determine that. In addition, a limitation of the questionnaires in this study is the use of different time points in the questions: last month, last 3 months and ever. Therefore, it is difficult to link the results of these questions to each other. Finally, in this study employment specialists were aware that they were participating in a study on improving work participation outcomes of people with MHI. This may have lead to the Hawthorne effect [51], i.e. employment specialists could have become more motivated to support people with MHI because of this extra awareness.

### Implications for Research and Practice

Results of previously published RCT showed that the stigma awareness intervention was highly effective, as about twice as many clients in the experimental group found and retained paid employment after 6 (51% versus 26%) and 12 months (49% versus 23%) respectively. Moreover, clients in the intervention group were significantly more satisfied with the support received than in the control group [30]. This indicates that more attention towards MHI stigma awareness and the disclosure dilemma contributes to improved (and sustainable) labor participation and satisfaction with support from employment specialists. It is recommended that future research evaluate the effects of the intervention more specifically on changes in disclosure decisions and subsequent outcomes. The findings of current study have major implications for practice, as this suggests that as found in current study that implementing this feasible, inexpensive and relatively simple stigma awareness intervention in municipal practice, could possibly double the employment rates of unemployed people with MHI. Improving the employment outcomes of people with MHI, will both have personal positive effects, e.g. better health and wellbeing [1, 6], as well as societal benefits, such as lower societal costs. However, the findings of the current study show that many employment specialists found the tools and training not so important and that the large majority did not use the tool anymore 1 year after the trainings. Therefore, it is highly important to continue to educate employment specialists about the importance of the topic, tools and training.

## Conclusion

This process evaluation showed that the implementation of a stigma awareness intervention was feasible and did increase stigma awareness in both clients and employment specialists. Experiences with the intervention were mainly positive, as 87% of the clients would recommend the CORAL.NL tool to others. Considering the intervention about doubled the number of clients who found and retained paid employment and lead to clients being more satisfied with the support received from their employment specialists [30] it is highly important to increase awareness and motivate employment specialists use the tools and address the topics of stigma and disclosure with their clients. Findings of the present study showed that the low interest employment specialists had in the topic is a concern and priority for future studies.

## Supplementary Information

Below is the link to the electronic supplementary material.

Supplementary material 1 (PDF 398.6 kb)

Supplementary material 2 (PDF 252.4 kb)

Supplementary material 3 (DOCX 17.2 kb)

Supplementary material 4 (PDF 299.7 kb)

Supplementary material 5 (DOCX 14.6 kb)

## Data Availability

The datasets generated during and/or analyzed during the current study are available from the corresponding author on reasonable request.

## Abbreviations

RCT: Randomized Controlled Trial
CORAL: Conceal or Reveal
MHI: Mental health issues/Mental illness
