# Supplementary material for: Improving Work Participation Outcomes Among Unemployed People with Mental Health Issues/Mental Illness: Feasibility of a Stigma Awareness Intervention
Source: J Occup Rehabil. 2023 Oct 25;34(2):447–60. doi: 10.1007/s10926-023-10141-3 (PMC11180002; doi:10.1007/s10926-023-10141-3)
Supplement: Supplementary file 3 — Supplementary material 3 (DOCX 17.2 kb) [file 10926_2023_10141_MOESM3_ESM.docx]

Manuscript

**Improving work participation outcomes among unemployed people with mental health issues/mental illness: Feasibility of a stigma awareness intervention**

**Appendix 2:** learning goals and components of the training

|  | **Training components** | **Learning goals of training** |
| --- | --- | --- |
| Training session I | - Informative presentation ‘What is stigma and why it is a problem for labor participation?’ - Discussion about statements on the attitudes of managers. - Interview with a mental health advocate with lived experience. - Discussion about own attitudes and acting. - Explaining how to use the CORAL.NL tool. - Formulating personal learning goals. | - - Create/increase awareness of stigma and discrimination in the work environment:  1. What stigma is and how does it work, what are the effects? 2. Increase awareness into stigma and discrimination by employers/managers; 3. Increase awareness into the effects of own attitudes, personal prejudices and actions; 4. Increase awareness into negative effects of disclosure during job applications.  - Learn to use the CORAL.NL tool in conversations with clients, without influencing too much. - Motivate and enthuse for this theme and new working method. |
| Training session II | - Evaluating the progress of recruiting participants - Displaying film with stories of workers with lived experience, created for the purpose of this training. - Improving conversation skills and using the CORAL.NL tool. - Role play to practice the conversation between employment specialist and client about disclosure of mental health issues/mental illness. - Discussion about statements on the effectiveness of a disclosure decision aid. - Formulating personal learning goals. | - - Improve skills for working with CORAL.NL:  1. What is going well? 2. What is going not so well? What are barriers? 3. For whom does it work or not? 4. When does it work or not? 5. What are reactions to CORAL.NL and/or the conversation about disclosure?    - Provide extra information for the successful use of the CORAL.NL tool and on how to enter into a conversation about disclosure.    - Increase awareness of stigma and discrimination in the work environment: 6. Increase awareness into stigma and discrimination by employers/managers. 7. Increase awareness into how stigma can be experienced/what it does to people. |
| Training session III | - Informative presentation about the attitudes of different stakeholder groups towards disclosure of mental health issues/mental illness. - Improving conversation skills and using CORAL.NL. - Role play with a mental health advocate with lived experience to practice the conversation about the disclosure-dilemma. | - - Discussion of the content of CORAL.NL and how to work with the CORAL.NL tool:  1. Topics covered in CORAL.NL. 2. Learn to work with CORAL.NL.  - Increase awareness into stigma and discrimination by employers/managers. - Increase awareness into how the disclosure dilemma can be experienced/what it does to people. |
