# Supplementary material for: Improving Work Participation Outcomes Among Unemployed People with Mental Health Issues/Mental Illness: Feasibility of a Stigma Awareness Intervention
Source: J Occup Rehabil. 2023 Oct 25;34(2):447–60. doi: 10.1007/s10926-023-10141-3 (PMC11180002; doi:10.1007/s10926-023-10141-3)
Supplement: Supplementary file 5 — Supplementary material 5 (DOCX 14.6 kb) [file 10926_2023_10141_MOESM5_ESM.docx]

| **Appendix 3**. Overview of respondents with one or more self-reported diagnoses. | | | | |
| --- | --- | --- | --- | --- |
|  | | **N(%) participants who did not have a diagnosis or did not know the diagnosis** | **N (%) participants with diagnosis for one mental health issue/illness** | **N (%) participants with diagnosis for two or more mental health issues/illness** |
| Total | | 18 (23.7%) | 20 (26.3%) | 38 (50.0%) |
|  |  |  |  |  |
| Differentiated in | |  |  |  |
|  | No diagnosis | 11 (61.1%) | - | - |
|  | Did not know the diagnosis | 7 (38.9%) | - | - |
|  | Anxiety | - | 2 (10.0%) | 4 (10.5%) |
|  | Attention deficit (hyperactivity) disorder | - | 0 (0%) | 11 (28.9%) |
|  | Autism spectrum disorder | - | 0 (0%) | 14 (36.8%) |
|  | Bipolar disorder | - | 1 (5.0%) | 1 (2.6%) |
|  | Burnout, overload, stress | - | 4 (20.0%) | 8 (21.1%) |
|  | Depression | - | 4 (20.0%) | 16 (42.1%) |
|  | Personality disorder | - | 3 (15.0%) | 8 (21.1%) |
|  | Psychotic disorder | - | 0 (0%) | 3 (7.9%) |
|  | Posttraumatic Stress Disorder | - | 5 (25.0%) | 7 (18.4%) |
|  | Other | - | 1 (5.0%) | 6 (15.8%) |
